# Tryptophanase Expressed by *Salmonella* Halts Breast Cancer Cell Growth In Vitro and Inhibits Production of Immunosuppressive Kynurenine

**DOI:** 10.3390/microorganisms11051355

**Published:** 2023-05-22

**Authors:** Eljoie Anice Cada Hababag, Allea Cauilan, David Quintero, David Bermudes

**Affiliations:** 1 Department of Biology, California State University Northridge, Northridge, CA 91330, USA; eljoie-anice.hababag@csun.edu (E.A.C.H.); allea.cauilan@csun.edu (A.C.); 2 Los Angeles Medical Facility, Los Angeles, CA 90027, USA; dquintero2@dhs.lacounty.gov

**Keywords:** tryptophanase, kynurenine, *Salmonella*, VNP20009, amino acid depletion

## Abstract

Tryptophan is an essential amino acid required for tumor cell growth and is also the precursor to kynurenine, an immunosuppressive molecule that plays a role in limiting anticancer immunity. Tryptophanase (TNase) is an enzyme expressed by different bacterial species that converts tryptophan into indole, pyruvate and ammonia, but is absent in the *Salmonella* strain VNP20009 that has been used as a therapeutic delivery vector. We cloned the *Escherichia coli* TNase operon *tnaCAB* into the VNP20009 (VNP20009-*tnaCAB*), and were able to detect linear production of indole over time, using Kovács reagent. In order to conduct further experiments using the whole bacteria, we added the antibiotic gentamicin to stop bacterial replication. Using a fixed number of bacteria, we found that there was no significant effect of gentamicin on stationary phase VNP20009-*tnaCAB* upon their ability to convert tryptophan to indole over time. We developed a procedure to extract indole from media while retaining tryptophan, and were able to measure tryptophan spectrophotometrically after exposure to gentamicin-inactivated whole bacterial cells. Using the tryptophan concentration equivalent to that present in DMEM cell culture media, a fixed number of bacteria were able to deplete 93.9% of the tryptophan in the culture media in 4 h. In VNP20009-*tnaCAB* depleted tissue culture media, MDA-MB-468 triple negative breast cancer cells were unable to divide, while those treated with media exposed only to VNP20009 continued cell division. Re-addition of tryptophan to conditioned culture media restored tumor cell growth. Treatment of tumor cells with molar equivalents of the TNase products indole, pyruvate and ammonia only caused a slight increase in tumor cell growth. Using an ELISA assay, we confirmed that TNase depletion of tryptophan also limits the production of immunosuppressive kynurenine in IFNγ-stimulated MDA-MB-468 cancer cells. Our results demonstrate that *Salmonella* VNP20009 expressing TNase has improved potential to stop tumor cell growth and reverse immunosuppression.

## 1. Introduction

Uncontrolled growth of cancerous tissues requires a supply of metabolic substrates [1,2,3,4]. In addition to excessive consumption of glucose, known as the Warburg effect, cancer cells are highly dependent upon amino acids [1,2,4,5]. While essential amino acid dependence is strictly required, normally non-essential amino acids may also be conditionally required by cancerous tissues due to down-regulation of amino acid biosynthesis, whereby the cells become auxotrophic through genetic reprogramming [2,3].

The depletion of amino acids using systemically delivered enzymes as an approach to cancer therapy is an ongoing area of investigation [6,7,8]. L-asparaginase has been approved for use in cancer therapy and is widely used in the treatment of acute lymphoblastic leukemia (ALL). Other amino acid degrading enzymes, arginine deaminase and methionase, have also undergone investigation [8]. The different enzymes studied are targeted either for degradation of conditionally required amino acids (asparagine, arginine and glutamine) or an essential amino acid (methionine). It has recently been shown that L-asparaginase combined with the autophagy blocker chloroquine enhances apoptosis [8], which demonstrates the potential of this approach for developing combination therapies. 

In addition to fueling cancer cell growth, amino acid metabolism is also linked to immunosuppression [3,9]. Indoleamine 2,3-dioxygenase (IDO) and the closely related enzyme tryptophan 2,3-dioxygenase (TDO) utilize tryptophan as the substrate in the first reaction toward generating kynurenine, a molecule suppressive to T-cells that plays a role in both cancer cell immune evasion and preventing rejection during pregnancy. Inhibitors of IDO have shown promise in treating several types of cancers, including melanoma, breast, pancreatic and colon cancers [10,11].

The therapeutic potential of targeting tryptophan metabolism for the treatment of cancer is well-recognized [12], as well as the rational for inhibiting IDO [13], and is considered an important therapeutic target [14]. Tryptophan is an amino acid essential to all human cells. Because tryptophan is both essential and a precursor to immune-suppressive kynurenine, we sought to determine if tryptophan depletion would alter the growth of human triple negative breast cancer cells. We engineered *Salmonella* VNP20009 [15] to constitutively express tryptophanase, using the tryptophan operon from *Escherichia coli*. 

Tumor-targeted bacteria is an area of interest in delivering therapeutics through intratumoral gene expression of tumor-localized bacteria [16,17,18]. Several bacteria are under investigation for their ability to selectively localize within tumors following systemic administration, including from the genera *Clostridium, Listeria, Escherichia* and *Salmonella*. Currently, the *Salmonella* strain VNP20009 and its derivative (TAPET-CD) remain the most well investigated in humans [19,20,21,22], and VNP20009 has also been investigated in a veterinary trial [23]. In human studies, the bacteria were shown to be safe, and to target tumors in some patients. However, there is a need to improve antitumor activity. Asparaginase delivered by *Salmonella* has been shown to increase the activity of the bacteria in suppressing tumor cells [24]. In this study, we sought to investigate *Salmonella* VNP20009 expressing tryptophanase.

## 2. Materials and Methods

Bacterial Strains Used in the Study. *Escherichia coli* DH5α F^–^ φ80*lac*ZΔM15 Δ(*lac*ZYA-*arg*F)U169 *rec*A1 *end*A1 *hsd*R17(r_K_^−^, m_K_^+^) *pho*A *sup*E44 λ^–^*thi*-1 *gyr*A96 *rel*A1, *E. coli* MG1655 (*λ^-^ rph-1*, Coli Genetic Stock Center #6300), *Salmonella enterica* serotype Typhimurium JR501 (*hsdSA29 hsdSB121 hsdL6 metA22 metE551 trpC2 ilv-452 H1-b H2-e,n,x (cured of Fels 2) fla-66 nml rpsL120 xyl-404 galE719* (*Salmonella* Genetic Stock Center, Calgary, Canada, strain 1593; [25] and VNP20009 (American Type Culture Collection (ATCC; Manassas, VA, USA) strain 202165 (a.k.a. 41.2.9 or YS1646; [15]. The complete genome of this strain has been determined [26]. 

Bacterial cultures were grown overnight in LB-0 (LB media without salt [27], consisting of 1% tryptone, 0.5% yeast extract or LB-0 plates containing 1.5% agar. Ampicillin at 100 μg/mL ampicillin (Amp_100_) was used for plasmid containing strains. The colony-forming units (CFU) were determined by plating serial dilutions to LB-0 and counting the colonies the following day. The optical density of the culture was determined by the optical density at 600 nm (OD_600)_ of a 1:10 dilution, and the OD_600_:CFU result was used to estimate the CFU for subsequent experiments based upon OD_600_. 

Cloning Tryptophanase and Detecting Tryptophanase Activity. In *E. coli,* tryptophanase is encoded by an operon that consists of transcribed leader region, *tnaC* (also known as *tnaL*), and two larger structural genes, *tnaA*, which encode the degradative enzyme, and *tnaB*, which is involved in tryptophan transport. DNA oligonucleotide primers used in the study are shown in Table 1. The tryptophanase operon of *E. coli* MG1655 (Genbank U00096.2) was amplified with the primers p*tna*_*Kpn*I_F1 containing a *Kpn*I site and p*tna*_*XbaI*_R1 containing an *Xba*I site, using KOD hotstart mastermix (EMD Millipore, Billerica, MA, USA). The PCR product was cloned into the *Kpn*I and *Xba*I sites pTrc99a [28] (GenBank U13872.1) using rapid ligation (Fermentas, Vilnius, Lithuania) in order to generate pTrc99a-*tnaCAB*. Because the initial cloning contained all empty vectors, the ligation was concentrated and desalted (Zymoclean, Zymo Research, Irvine, CA, USA) and directly transformed by electroporation [29] into *Salmonella* JR501. This facilitated screening for enzyme activity, which was not possible in the *E. coli* DH5α because the strain is already positive for tryptophanase. A colony lift was performed with 85 mm sterilized Whatman Grade 1 filter paper (ThermoFisher, Waltham, MA, USA) and tryptophanase activity detected as green spots by exposing the entire filter to 1 mL of the indole spot test (RapID Spot, Remel, Lenexa, KS, USA). A corresponding green colony was chosen, and the DNA sequence of the insert determined in both strands, using the primers pAraF1, pTrcR2, For_1 to For_5, and Rev_1 to Rev_5. The empty vector pTrc99a plasmid DNA from JR501 and the pTrc99a plasmid with the tryptophanase operon were independently transformed to VNP20009 to generate the VNP20009 carrying the empty pTrc99a vector (VNP20009-EV) and the VNP20009 with the *tna*-expression plasmid (VNP20009-*tnaCAB*). 

**Figure 1:**
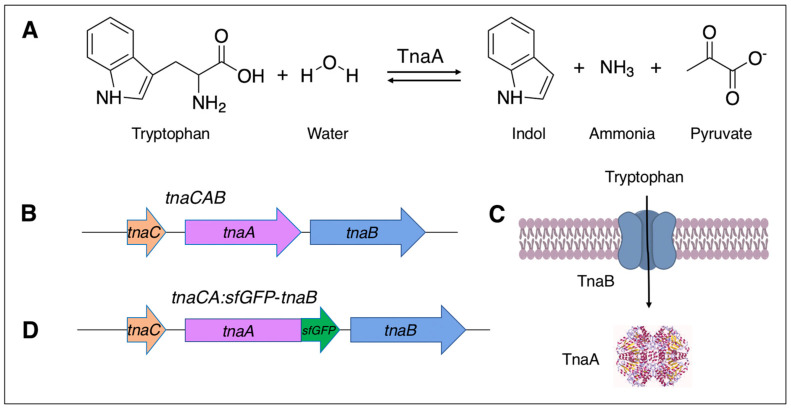
Tryptophanase. (**A**) The enzymatic reaction and products of tryptophanase. (**B**) The tryptophanase operon *tnaCAB*, which consists of the regulatory element *tnaC*, the enzymatic encoding component *tnaA* and the component encoding the tryptophan transporter *tnaB*. (**C**) A generalized two-component organization, the *TnaB* and *TnaA*. Tryptophanase homotetramer ribbon diagram from CC File:4w1y.jpg. (**D**) The tryptophanase operon with *sfGFP* encoding superfold green fluorescent protein (sfGFP) fused to *tnaA*. The chemical structures were generated with ChemDraw (Perkin Elmer, Waltham, MA, USA).

A TnaA fusion with superfold GFP (*tnaA*:*sfGFP*) was generated by overlapping PCR. The first reaction utilized the same forward cloning primer p*tnaCA*_*Kpn*I_F1 and p*tnaA*_nostop_R1. The second reaction primers were p*tnaA*_nostop_ W*sfGFP*_F1 and p*sfGFP*_nostop*Xba*I_R1. A total of 1 μL of each of the two products was used as the co-templates, with only the outer primers, p*tnaCA*_*Kpn*I_F1 and p*sfGFP*_nostop*Pac*I*Xba*I_R1, used for amplification; the product was cloned into pAra99a [30] and in the *Kpn*I and *Xba*I restriction sites and then moved to pTrc99a to result in pTrc99a*-tnaCA:sfGFP*. *tnaB* was then subcloned from the original construct into the *Pac*I and *Xba*I sites of pTrc99a*-tnaCA:sfGFP*, to result in pTrc99a*-tnaCA:sfGFP*-*tnaB*. The clones were then sequentially transformed to JR501 and then VNP200009. The *tna* operon and *tna* operon with the *tnaA:sfGFP* fusion are shown in Figure 1.

Determination of intracellular localization of tryptophanase. The VNP20009-EV and VNP20009-*tnaCA:sfGFP-tnaB*, as well as their *E. coli* DH5α counterparts, were cultured in LB-0 broth with 100 μg/mL ampicillin at 37 °C overnight, in a rotator. The next day the bacteria were subcultured at an initial OD_600_ of 0.1 and allowed to double twice to OD_600_ 0.4. A 1% agarose pad was generated on a microscope slide by pipetting 120 μL of hot 1% agarose in tris acetate EDTA buffer (TAE) onto a microscope slide and placing a coverslip on top. The pad was allowed to cool and solidify, and the coverslip removed. A sample of the culture was then added to the pad, and the coverslip replaced. The bacteria were imaged with a Zeiss Axio Imager M1, with photographs taken with a Hamamatsu ORCA-ER camera using differential interference contrast (DIC) light microscopy and with GFP fluorescence microscopy. The images were merged using AxioVision software 4.6.3.0.

Quantitative Indole Determinations. High-purity indole (99+%, ACROS Organics, Geel, Belgium) dissolved in 70% ethanol was used to create a standard curve (from 0 to 100 μM) in LB-O culture media. The 250 μL samples were mixed with an equal volume of Kovács reagent (isoamyl alcohol, para-dimethylaminobenzaldehyde and concentrated HCl; Remel) to generate a fuchsia-colored complex with para-dimethylaminobenzaldehyd. The samples were vortexed for 10 sec, centrifuged 17,000× *g* for 2 min, 100 μL of the supernatant transferred to a microtiter plate, and the absorbance determined at 570 nm using SpectraMax M3 (Molecular Devices, Sunnydale, CA, USA), using SoftmaxPro 6.2. Data from SoftmaxPro were exported to Microsoft Excel for data analysis. 

Tryptophan Conversion by VNP20009-EV and VNP20009-*tnaCAB* to Indole. L-Tryptophan (99%, Alfa Aesar, Ward Hill, MA, USA) was added to 0.9% saline to generate a 0.5 mM stock concentration and filter sterilized through a 0.22 μm filter (Nalgene, Rochester, NY, USA). Bacteria were pelleted and resuspended in saline three times, and 1 × 10^8^ CFU/mL used for the tryptophan to indole conversion studies, with 100 μM tryptophan.

Determining the Relative Rates of Tryptophanase Activity with and Without Antibiotic Treatment. Tryptophan conversion with and without 100 μg/mL gentamycin [31] using 1 × 10^8^ CFU/mL was assessed for VNP20009-EV and VNP20009-*tnaCAB*. Samples were taken at 0, 15, 30 and 60 min, and the indole concentration was quantified as described above. Based on this study we added gentamycin after bacterial growth in order to stop further growth in subsequent studies when added to the tissue culture media and thereby diminish other effects that bacterial growth could cause, including the depletion of other amino acids.

Separation of Indole from Tryptophan. Tryptophan and indole share the same heterocyclic aromatic rings, which makes it difficult to distinguish each of them in the presence of the other. We investigated whether indole could be separated from tryptophan by organic extraction in order to quantify the tryptophan residue. In preliminary studies, indole was found to be removed from aqueous solution by extraction with chloroform, while tryptophan remained in the aqueous phase. Indole was extracted from 1.5 mL of the sample by the addition of 0.5 mL chloroform, vortexed for 10 s, centrifuged at 17,000× *g* for 1 min, and the aqueous phase transferred to a new tube for a total of three successive extractions.

Measurement of Tryptophan Following Extraction of Indole. Tryptophan in tris buffered saline (TBS) pH 7.4 was scanned with the Spectramax M3 using matched quartz cuvettes of between 200 and 300 nm, and found to have peak adsorptions at 218 and 280 nm, with 218 nm having the higher readings. Following chloroform extraction, the spectra shifted slightly but the peaks remained at 218 and 280 nm. OD_218_ was determined for a standard curve, ranging from 0 to 50 μM tryptophan. When samples had concentrations greater than 50 μM, they were diluted to give a reading below that corresponding to 50 μM and their concentration calculated using the dilution factor.

Tryptophan Depletion. Tryptophan was adjusted to 80 μM final concentration in TBS in order to match the concentration in DMEM cell culture media, and VNP20009-*tnaCAB* bacteria ranging from 1 × 10^9^–1 × 10^5^ with 100 μg/mL gentamycin were added to the tryptophan solution and incubated for 4 h at 37 °C. After 4 h, the bacteria were removed by centrifugation at 17,000× *g* for 1 min, and 1.5 mL of the supernatant was extracted with chloroform and measured for tryptophan at OD_218_, as described above. 

Tryptophan Depletion of Tumor Cell Culture Media. To generate tryptophan-depleted media based upon the 4 h data, 1 × 10^8^ CFU/mL saline-washed bacteria was added to DMEM with 10 % (*v*/*v*) fetal bovine serum (FBS) with 100 μg/mL of gentamycin, without phenol red. The culture medium was allowed to incubate for 4 h at 37 °C, in a rotator. The bacteria were then removed by centrifugation at 8000× *g* for 10 min, and then sterilized by passage through a 0.22 μm surfactant-free cellulose acetate (SFCA) sterile filter (Thermo Scientific, Swedesboro, NJ, USA). In preliminary experiments, media with phenol red did not change color, and no change in pH was detected due to the presence of the bacteria. This same process was used for depleted media plus tryptophan, which was added back at 80 μM. The entire process was also performed with the VNP20009-EV strain, for comparison. The other group was one with an 80 μM mixture of indole, ammonia and pyruvate to control for the potential contribution of these metabolites.

Tumor Cells. MDA-MD-468 human breast carcinoma ATCC HTB-132 were grown in Dulbecco’s Modified Eagle Medium (DMEM) containing 4.5 g/L glucose (Gibco, Grand Island, NY, USA) with 10% (*v*/*v*) added fetal calf serum (Gibco) plus pyruvate (Gibco) and glutamate (Gibco). The cell line was authenticated at the University of Arizona Genetics Core (Tucson, AZ, USA). Cells were trypsinized (0.5% trypsin 0.2% EDTA, Sigma-Aldrich, St. Louis, MO, USA) for seeding the tissue culture flasks (Corning, Corning, NY, USA) and culture well plates (Falcon, Lexington, TN, USA). 

Effect of Conditioned Tissue Culture Media on Tumor Cell Growth. We compared five different groups of MDA-MB-468 tumor cells consisting of untreated media, media with the 80 μM of combined indole, ammonia and pyruvate (the maximum theoretical concentrations for conversion of 80 μM of tryptophan in the media), VNP20009-EV conditioned media, VNP20009-*tnaCAB* conditioned media, and VNP20009-*tnaCAB* with 80 μM of tryptophan added back after conditioning. Cells were seeded in 6-well plates with two mL of each of the above, with all media having been normalized for dilutions with saline and the small amount of ethanol (0.2%) required for the group containing indole. 

Effect of Conditioned Tissue Culture Media on Tumor Cell Growth and Kynurenine Production. Kynurenine was measured in the same groups as above. Cells were stimulated with interferon gamma (IFNγ; Pierce Biotechnology, Rockford, IL, USA) 1000 u/mL, as described by [32] for the MDA-MB-468 cell line. Kynurenine was measured by ELISA (Rocky Mountain Diagnostics, Colorado Springs, CO, USA). In order to normalize for any differences in growth among the groups, the resazurin (Alamar Blue) viability assay (R&D Systems, Minneapolis, MN, USA) was performed, and the results divided by the relative fluorescence 560/590 nm, using a SpectraMax M3 (Molecular Devices, Sunnydale, CA, USA) running SoftmaxPro 6.2. The absorbance value from a media-only group was subtracted from the results and used to generate a standard curve.

Statistical Analysis. Data from SoftmaxPro were exported to Microsoft Excel for initial data analysis, and then imported into Prism 6.0 (GraphPad) for statistics using one-way ANOVA and two-tailed *t*-tests. 

## 3. Results

### 3.1. Cloning of Tryptophanase and Detecting Tryptophaase Activity in Salmonella VNP20009 

**Figure 2:**
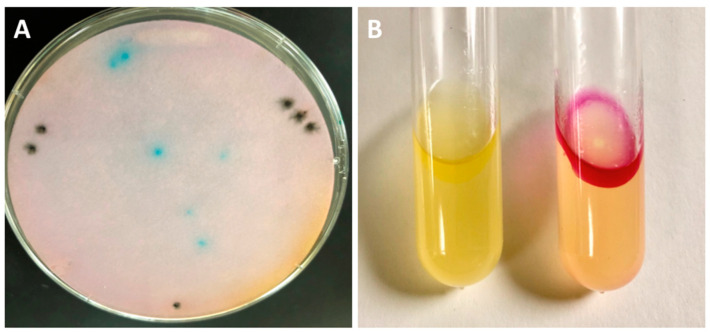
Detecting tryptophanase in transformed *Salmonella*. (**A**) *Salmonella* JR501 was directly transformed with the trytophanase operon ligation, colony lifted, and the filter paper soaked in rapid spot assay. Green (positive) colonies were rapidly visible. Note: the black spots represent guide marks for alignment with the original plate that were made with a needle dipped in India ink, which were used to align with the corresponding colonies. (**B**) *Salmonella* VNP20009 transformed with an empty pTrc99a (VNP20009-EV) vector (left) and the pTrc99a-tnaCAB (right) tested with Kovács reagent, showing a positive (fuchsia) color change.

Direct transformation into *Salmonella* JR501 resulted in the ability to detect tryptophanase activity using the rapid spot assay on a colony lift (Figure 2A). A positive (green) clone from the corresponding plate was plasmid prepped and transformed to DH5*α* for DNA sequencing, which showed the complete tryptophanase operon had successfully been cloned. When the DNA from *Salmonella* JR501 was then transformed to *Salmonella* VNP20009 and tested for indole production using Kovács reagent compared to *Salmonella* VNP20009 carrying an empty vector (VNP20009-EV), the result showed a strong positive (fuchsia) only for the tryptophanase-carrying strain (Figure 2B).

### 3.2. Intracellular Localization of Tryptophanase in Salmonella VNP20009 

**Figure 3:**
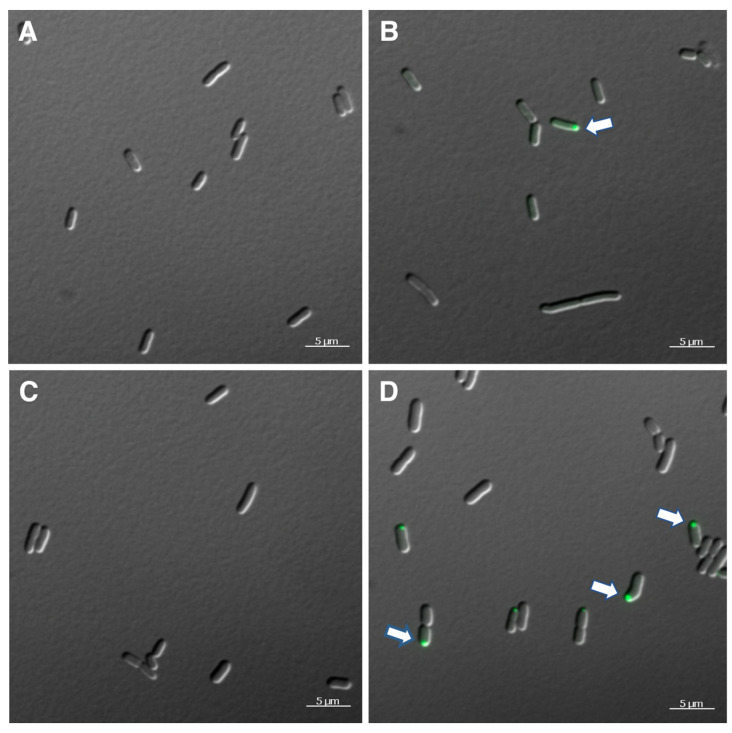
Light and fluorescent microscopy showing intracellular localization of tryptophanase. (**A**) *E. coli* containing the pTrc99a empty vector. (**B**) *E. coli* containing the pTrc99a-tnaCAB. (**C**) *Salmonella* VNP200009 containing the pTrc99a empty vector. (**D**) *Salmonella* VNP20009 pTrc99a-tnaCAB. Magnification bars, 5 μm. White arrows indicate areas of intracellular localization.

Light and the fluorescent microscopic images of the control *E. coli* with the empty vector and the TnaA:sfGFP fusion clearly show that the *E. coli* possess no significant autofluorescence in the GFP channel, and that the GFP fluorescence is localized to a single, small, spherical intracellular location (Figure 3A,B). This is consistent with the polar body localization of tryptophanase previously reported for *E. coli* [33]. Light and fluorescent microscopic images of the control *Salmonella* VNP20009 with the empty vector and the TnaA:sfGFP fusion also clearly show that *Salmonella* possesses no significant autofluorescence in the GFP channel, and that the GFP fluorescence is localized to similar, small. spherical intracellular locations visible in some but not all of the cells (Figure 3C,D). This is also consistent with polar body localization. As *E. coli* and *Salmonella* are closely related and are both known to have microcompartments [34], it appears that the *E. coli* tryptophanase microcompartment targeting signal is functional in *Salmonella*. 

### 3.3. Quantitative Indole Determinations and Tryptophan Conversion by VNP20009-EV and VNP20009-tnaCAB 

**Figure 4:**
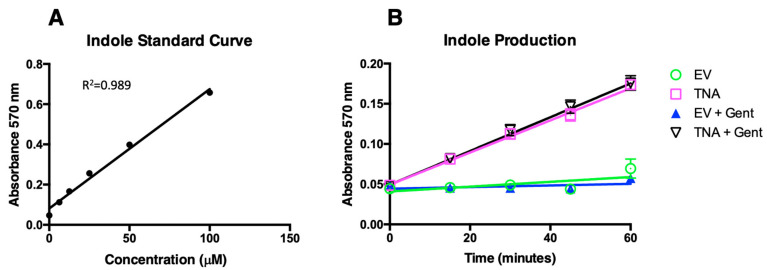
Bacterial conversion of tryptophan to indole. VNP20009-*tnaCAB* (TNA) was compared with VNP20009 carrying the empty vector (EV + Gent) with and without treatment with 100 μg/mL gentamycin (Gent). (**A**) An indole standard curve from 0 to 100 μM read for absorbance at 570 nm. (**B**) A 60-min time course detecting conversion of tryptophan to indole using Kovács reagent and measuring absorbance at 570 nm.

We found that measurement of the Kovács reagent resulted in a linear curve of over 0 to 100 μM indole. Treatment with gentamycin had no observable effect on tryptophanase activity (Figure 4). We calculated the enzymatic rate associated with the fixed amount of the bacteria (1 × 10^8^ CFU/mL) using 100 μM tryptophan as the substrate, and found that 1 × 10^8^ CFU/mL converted 266 (±8.26%) nM per min. 

### 3.4. Separation of Indole from Tryptophan and Measurement of Tryptophan 

**Figure 5:**
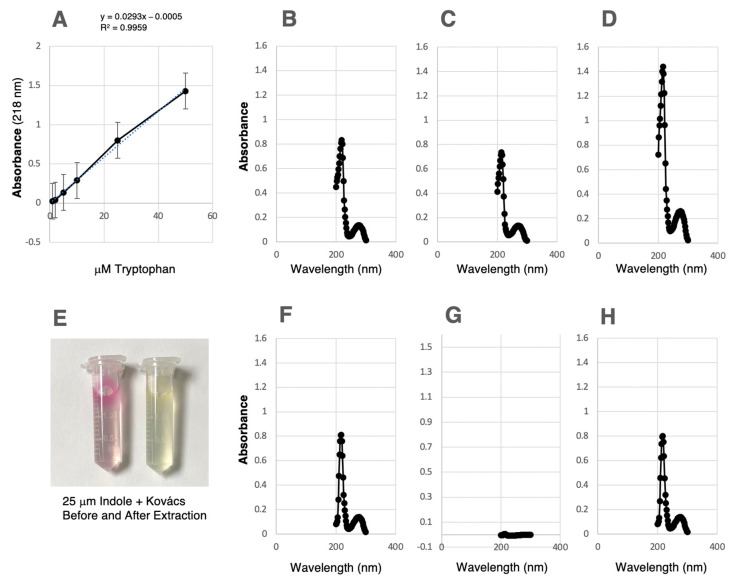
Separation of indole from tryptophan and measurement of tryptophan. (**A**) A tryptophan standard curve ranging from 1 to50 μM measured by absorbance at 218 nm. (**B**) Absorbance from 200 to 300 nm of 25 μM tryptophan. (**C**) Absorbance from 200 to 300 nm of 25 mm tryptophan. (**D**) Absorbance from 200 to 300 nm of 25 μM tryptophan plus 25 μm indole. (**E**) The reaction of Kovács reagent with 25 μM indole without (left) and with (right) prior extraction with chloroform. (**F**) Absorbance from 200 to 300 nm of 25 μM tryptophan extracted with chloroform. (**G**) Absorbance from 200 to 300 nm of 25 μM indole extracted with chloroform. (**H**) Absorbance from 200 to 300 nm of 25 μM tryptophan plus 25 μM indole extracted with chloroform.

A tryptophan standard curve ranging from 1 to 50 mM was generated using the peak absorbance of 218 nm, which resulted in a linear curve with an R^2^ of 0.999 (Figure 5A). The detection limit (DL) was calculated as 3.3 × σ/slope where sigma s is the standard deviation of the curve, with the DL determined to be 0.84 μM. Tryptophan and indole showed very similar scanning spectra between 200 and 300 nm (Figure 5B,C). Similarly, the combination of the two in the same solution gave a much larger, although not completely additive, curve (Figure 5D). When indole was added at 25 mM and then tested with Kovács reagent, a fuchsia color quickly appeared (Figure 5E, left), but when indole was added at 25 mM followed by extraction with chloroform, there was no color change with the addition of Kovács (Figure 5E, right). When tryptophan only was extracted with chloroform, the curve remained similar and had a very similar peak height (Figure 5F). However, when indole was extracted with chloroform, the peak was almost entirely eliminated (Figure 5G). When tryptophan was mixed with indole and then extracted, the remaining peak (Figure 5H) matched that of tryptophan in both the unextracted (Figure 5B) and the chloroform-extracted (Figure 5F) peaks. These data show that it is possible to separate indole from tryptophan and to quantitatively measure tryptophan.

### 3.5. The Ability of VNP20009-tnaCAB to Deplete Tryptophan 

**Figure 6:**
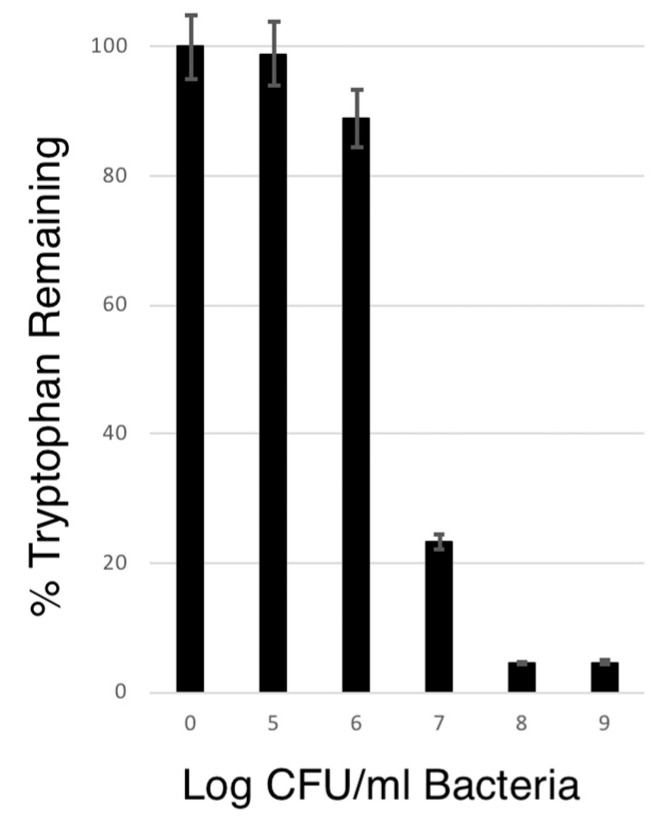
Depletion of Tryptophan. Fixed amounts of bacteria, ranging from 1 × 10^5^ to 1 × 10^9^ CFU/mL, were exposed to 80 μM tryptophan in TBS. After 4 h, the bacteria were removed by centrifugation followed by 0.2 μm filtration, and then measured for tryptophan using the method described above.

Using different amounts of bacteria, ranging from 1 × 10^5^ to 1 × 10^9^ CFU/mL for a fixed time of 4 h, we were able to show tryptophan depletion from Tris-buffered saline (TBS) pH 8.0. Our results showed that at least 1 × 10^6^ CFU/mL was required to have a measurable effect, and that in the 4 h period 1 × 10^8^ was enough to achieve maximum depletion, which was 93.9% ± 0.44%, with 95% confidence. The results are shown in Figure 6.

### 3.6. Effect of Media Depletion of Tryptophan

#### 3.6.1. Tumor Cell Growth Alteration 

**Figure 7:**
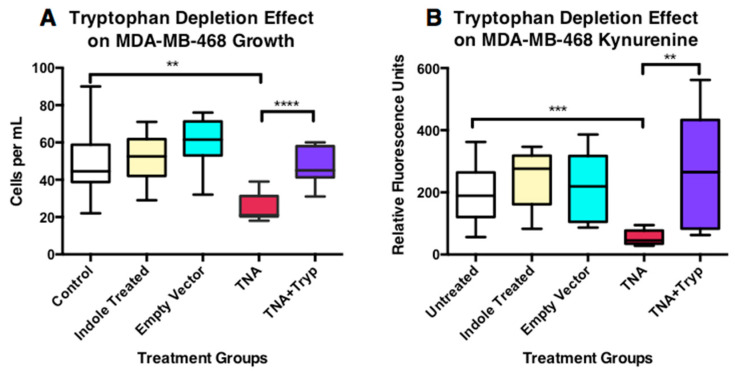
Effects of Tryptophan Depletion on Tumor Cell Growth and Production of Kynurenine. (**A**) The relative effects of various treatments on cell growth at three days. The groups consisted of (1) the untreated control, (2) media to which the 80 μM indole cocktail had been added, (3) the empty vector, which consisted of media treated with VNP20009 containing the pTrc99a plasmid without any insert, (4) tryptophanase (TNA)-treated media, and (5) tryptophanase-treated media with 80 μM tryptophan added back. The indole cocktail showed no statistically significant effect on cell growth, although a slight increase was noted. The empty vector treatment also showed no statistical effect on cell growth, although again, a slight increase was noted. The tryptophan-depleted (TNA) group was significantly reduced in its relative cell growth. The reduction in cell growth was restored by the re-addition of 80 μM tryptophan to TNA-depleted media, and was highly significant. (**B**) The effects of various treatments on production of kynurenine on IFNγ stimulated MDA-MB-468 cells at three days. The treatments represent the same as those in panel A, except that IFNγ was added at 1000 u/mL. The indole cocktail showed no statistically significant effect on kynurenine production. Empty vector treatment also showed no statistical effect on the production of kynurenine. The TNA-treated media resulted in a significant reduction in its production of kynurenine, which was also restored by the re-addition of tryptophan to TNA-depleted media. Statistical significance is indicated with asterisks; ** = *p* ≤ 0.01, *** = *p* ≤ 0.001, **** = *p* ≤ 0.0001.

The comparison of five different MDA-MB-468 tumor cell groups is shown in Figure 7A. Both the indole-cocktail and empty-vector groups showed slight, but non-statistically significant, increases in cell counts. However, the tryptophanase-treated group (TNA) showed a sharp decline in cell count that was statistically significant. When tryptophan was added back to the tryptophanase-treated media, tumor cell growth was restored and showed a high degree of statistical significance. Since the indole cocktail only slightly increased growth, the decreased growth effect is not due to the production of the indole, ammonia and pyruvate metabolites. Likewise, the antibiotic-treated empty vector (media treated with VNP20009 containing the pTrc99a plasmid without any insert) showed no significant effect on tumor cell growth.

#### 3.6.2. Alteration in the Production of Kynurenine 

In the kynurenine assay, we recognized that the depletion of tryptophan could alter the cell growth, and thereby indirectly show a lower level of kynurenine production. We used the resazurin viability assay to normalize all the groups when we graphed kynurenine production (Figure 7B). We again saw little effect of the indole cocktail or the treatment of the empty vector. However, we observed a strong reduction in the production of kynurenine in the tryptophan-depleted media group. Kynurenine production was partially restored when tryptophan was added to the tryptophan-depleted media. The variability in this group was large and may reflect aspects of kynurenine production that are more stochastic than cell growth.

## 4. Discussion

We expressed tryptophanase in the *Salmonella* strain VNP20009. Enzymatic activity was shown by the production of indole detected with the indole spot test, Kovács reagent, and by the depletion of tryptophan. These bacteria generated a linear production of indole over a 1 h time period. Once the bacteria were grown, the addition of the antibiotic gentamycin did not inhibit enzyme activity, but stopped bacterial growth that might have influenced other aspects of the study, including depletion of other amino acids. The GFP-tagged tryptophanase was localized to polar bodies in some cells of the *E. coli* and *Salmonella* observed*,* which are known to have microcompartments [34]. The known microcompartment operons include proteins with identified microcompartment signals. Tryptophanase has previously been shown to localize to polar bodies [33] in *E. coli*, and it is therefore likely that the same signals are functional in *Salmonella*. We were able to separate indole from tryptophan and were able to measure the residual tryptophan. We found that 1 × 10^8^ CFU/mL was able to deplete approximately 94% of the tryptophan in 4 h. When we exposed MDA-MB-468 tumor cells to the depleted media, their growth was significantly inhibited. It is important to note that the control consisted of media exposed to the same parent *Salmonella* strain carrying the empty vector plasmid did not show that effect. We also noted that the equimolar indole cocktail (indole, ammonia and pyruvate) slightly increased growth, although it was not statistically significant. This could mean that in the tryptophanase-treated group, the depletion of tryptophan also overcomes this slight growth stimulation. Similarly, the depletion of tryptophan had a significant effect on inhibiting IFNγ-stimulated cells from producing kynurenine. These depletion experiments were performed using a controlled pH. It is known that tumors have heterogeneous pH ranges, which are often between pH 6.5 and 6.9 [35], and therefore, these results may not represent the results that would occur within tumors. However, bacteria localized in tumors remain for weeks on end, and tryptophanase from related species (*Vibrio*) remains at 80% activity down to pH 6.5 [36]. Thus, there is significant potential for an effect in vivo.

We have identified an approach to modifying bacteria with the potential to enhance antitumor activity that we believe merits future studies in murine models of cancer. Indoleamine 2,3-dioxygenase (IDO) is the first enzyme involved in the kynurenine pathway (KP). IDO initiates the metabolism of tryptophan to N-formylkynurenine, which is subsequently further metabolized to kynurenine, kynurenic acid, 3-hydroxykynurenine, 3-hydroxyantrhanilic acid and picolinic acid. These specific metabolites are important in immune tolerance, with IDO conversion to N-formylkynurenine being the rate-limiting step in their production [37,38,39]. Kynurenine is the best studied of these metabolites, and has been shown to be toxic to lymphocytes, and to induce CD4^+^ cells to differentiate into immunosuppressive T-regulatory cells [39]. 3-hydroxyanthranilic acid and quinolinic acid are known have selective effects on cells and induce cell death in Th1 cells (T-helper cell type 1) but not in Th2 cells (T-helper cell type 2) [40,41].

Amino acid catabolism causes immunosuppressive effects that promote tumor growth, while the depletion of amino acids is known to suppress tumor growth. Aspariginase (Erwinase and Crisantaspase), derived from *Erwinia chrysanthemi* and *E. coli*, respectively, are approved therapies used for the treatment of acute lymphoblastic leukemia (ALL). Methionase, an L-methionine-degrading enzyme from *Pseudomonas putida*, is another potent enzyme being explored as an anticancer treatment [42], and L-arginine deaminase and L-lysine α-oxidase are also being investigated as cancer therapies [6]. Interestingly, arginase is known to have both positive and negative effects on tumors, with tumor inhibition due to depletion, or immune inhibition through inhibiting NO production by macrophages [43,44], although its overall effect is positive. Other non-amino acid lytic enzymes such as thiaminase also exhibit strong anticancer activity, and are being explored for their therapeutic potential [45].

Because tryptophan is the catabolic substrate for IDO, expression of the *E. coli* tryptophanase, which degrades tryptophan to indole rather than n-formylkynurenine, has the potential to curtail the production of the downstream metabolites and their immunosuppressive activities. *Salmonella* VNP20009 expressing tryptophanase was demonstrated in our studies, which was facilitated in vitro by using the whole antibiotic-treated bacterial particles (WAT-BP) that retained tryptophanase enzymatic activity. We propose that the tumor-localization capacity of attenuated *Salmonella* strains that often results in the accumulation of greater than 1 × 10^9^ CFU/g tumor tissue [46,47] could make these strains ideal carriers for this therapeutic approach. We found these *Salmonella* were efficient at depleting tryptophan in cell culture media, which limited both cell growth and kynurenine production of the triple negative breast cancer cell line MDA-MB-468 in vitro. Our hypothesis is that in a murine model of triple negative breast cancer in immune-competent mice such as the syngeneic 4T1 model, we would not only expect increased antitumor activity, but increased intratumoral lymphocytes, as measured by increased CD8^+^ T-cells. This approach may synergize with epidermal growth factor receptor (EGFR)-targeted toxins [30], which have been shown to kill the MDA-MB-468 cell line that expresses the EGFR. This effect was shown to be further enhanced by the co-expression of a protease inhibitor [48]. Because amino acid degrading enzymes have also been coupled with the autophagy inhibitor chloroquine that is already known to enhance *Salmonella* therapy [49], exploring these therapeutic properties in combination is a promising avenue for in vivo studies. 

## 5. Conclusions

Tryptophanase was cloned from *E. coli* and expressed in the *Salmonella enterica* serotype Typhimurium strain VNP20009, which lacks naturally occurring tryptophanase. The tryptophanase was shown to localize into polar bodies in some cells, similar to *E. coli*. Tryptophanase produced a linear curve for indole production and was not altered by treatment with gentamycin. Treatment with gentamycin, an inhibitor of protein synthesis, stopped bacterial cell growth and therefore limited the depletion of other amino acids which it could otherwise have taken up and incorporated into proteins during the experimental time period. Indole could be separated (removed) from tryptophan containing media by extraction with chloroform, allowing the quantitative detection of residual tryptophan. Gentamycin-treated tryptophan expressing bacteria were effective at removing the majority of tryptophan for the media. When exposed to media depleted in tryptophan by gentamycin-treated VNP20009-*tnaCAB*, human MDA-MB-468 triple negative cancer cells showed significant decreases in both growth and in IFNγ-stimulated kynurenine. As kynurenine is an immunosuppressive molecule, limiting its production may improve antitumor immune responses. Tryptophanase, which is naturally occurring in other tumor-targeted bacterial strains such as *E. coli* Nissel 1917, may account for some of its endogenous antitumor activity. Tryptophanase engineered into the vector VNP20009, which was previously been studied in preclinical and human clinical trials where it was shown to target tumors, warrants further studies in murine models of triple-negative breast cancer. 

## Figures and Tables

**Table 1:** DNA oligonucleotide primers used in the study.

Primer #	Primer Name	Primer Sequence 5’-3’	Primer Restriction Enzyme Site(s) (Underlined in Sequence)
1	p*tnaCA*_*Kpn*I_F1	GATCGGTACCAGGAGGAATTCACCATGAATATCTTACATATATGTGTGACCTCAA	*Kpn*I
2	p*tnaB* _*XbaI*_R1	GATCTCTAGAGAAGGATTTAGCCAAATTTAGGTAACAC	*Xba*I
3	pAraF1	ACCTGACGCTTTTTATCGCA	
4	pTrcR2	CCGCCAGGCAAATTCTGT	
5	For_1	TTAGCCGAGTCAGTGAAAAA	
6	For_2	AGACGACAGCTTCTTTGATGTG	
7	For_3	GCCACTCTCTTACCCTACATCC	
8	For_4	CACTGCGGGAACGTCTTACT	
9	For_5	CAATCTTATTCCGGCGATTG	
10	Rev_1	AATCAGTACCGGAATATAGATTTGC	
11	Rev_2	CACATCAAAGAAGCTGTCGTCT	
12	Rev_3	CACATCAAAGAAGCTGTCGTCT	
13	Rev_4	ATATTGCCGTGGAAACCAAA-	
14	Rev_5	GCCAAATTTAGGTAACACGTTAAA	
15	p*tnaA*_nostop_R1	GCCAAATTTAGG TAACACGTTAAAGA	
16	p*tnaA*_nostop_ W*sfGFP*_F1	TCTTTAACGTGTTACCTAAATTTGGCATGGTGAGCAAGGGCGA	
17	and p*sfGFP* stop*Pac*I*Xba*I_R1	GACTTCTAGAGACTTTAATTAATTACTTGTACAGCTCGTCCATGC	*Xba*I, *Pac*I

## Data Availability

The data is available from the Principal Investigator (D.B).
